# Predictors of changing patterns of adherence to containment measures during the early stage of COVID-19 pandemic: an international longitudinal study

**DOI:** 10.1186/s12992-023-00928-7

**Published:** 2023-04-17

**Authors:** Yuen Yu Chong, Wai Tong Chien, Ho Yu Cheng, Demetris Lamnisos, Jeļena Ļubenko, Giovambattista Presti, Valeria Squatrito, Marios Constantinou, Christiana Nicolaou, Savvas Papacostas, Gökçen Aydin, Francisco J. Ruiz, Maria B. Garcia-Martin, Diana P. Obando-Posada, Miguel A. Segura-Vargas, Vasilis S. Vasiliou, Louise McHugh, Stefan Höfer, Adriana Baban, David Dias Neto, Ana Nunes da Silva, Jean-Louis Monestès, Javier Alvarez-Galvez, Marisa Paez Blarrina, Francisco Montesinos, Sonsoles Valdivia Salas, Dorottya Őri, Bartosz Kleszcz, Raimo Lappalainen, Iva Ivanović, David Gosar, Frederick Dionne, Rhonda M. Merwin, Andrew T. Gloster, Angelos P. Kassianos, Maria Karekla

**Affiliations:** 1 The Nethersole School of Nursing, Faculty of Medicine, The Chinese University of Hong Kong, New Territories, Hong Kong SAR, China; 2 Department of Health Sciences, European University Cyprus, 1516 Nicosia, Cyprus; 3 Psychological Laboratory, Faculty of Public Health and Social Welfare, Riga Stradiņš University, Riga, Latvia; 4 Kore University Behavioral Lab (KUBeLab), Faculty of Human and Social Sciences, Kore University of Enna, Enna, Italy; 5 Department of Social Sciences, School of Humanities and Social Sciences, University of Nicosia, Nicosia, Cyprus; 6 Department of Nursing, Cyprus University of Technology, Limassol, Cyprus; 7 Cyprus Institute of Neurology and Genetics, Nicosia, Cyprus; 8 Department of Psychological Counseling and Guidance, Faculty of Education, Hasan Kalyoncu University, Gaziantep, Turkey; 9 Department of Psychology, Fundación Universitaria Konrad Lorenz, Bogotà, Colombia; 10 Faculty of Psychology, University of La Sabana, Chía, Colombia; 11 School of Applied Psychology, University College Cork, Cork, Ireland; 12 School of Psychology, University College Dublin, Dublin, Ireland; 13 Department of Psychiatry II, Innsbruck Medical University, Innsbruck, Austria; 14 Department of Psychology, Babeş-Bolyai University (UBB), Cluj-Napoca, Romania; 15 ISPA - Instituto UniversitárioAPPsyCI - Applied Psychology Research Center Capabilities & Inclusion, Lisbon, Portugal; 16 Faculdade de Psicologia da Universidade de Lisboa, Lisbon, Portugal; 17 CICPSI - Centro de Investigação Em Ciência Psicológica. Alameda da Universidade, Universidade de Lisboa, Lisbon, Portugal; 18 LIP/PC2S Lab, University Grenoble Alpes, Grenoble, France; 19 Department of Biomedicine, Biotechnology and Public Health, University of Cadiz, Cadiz, Spain; 20 Instituto ACT, Madrid, Spain; 21 Department of Psychology, European University of Madrid, Madrid, Spain; 22 Department of Psychology and Sociology, University of Zaragoza, Zaragoza, Spain; 23 Department of Mental Health, Heim Pal National Pediatric Institute, Budapest, Hungary; 24 Private Practice, Poland; 25 Department of Psychology, University of Jyväskylä, Jyväskylä, Finland; 26 Clinic for Psychiatry, Clinical Center of Montenegro, Podgorica, Montenegro; 27 Department of Child, Adolescent and Developmental Neurology, University Children’s Hospital, University Medical Center Ljubljana, Ljubljana, Slovenia; 28 Département de Psychologie, Université du Québec À Trois-Rivières, Trois-Rivières, Québec, G9A 5H7 Canada; 29 Department of Psychiatry and Behavioral Science, Duke University, Durham, NC USA; 30 Division of Clinical Psychology and Intervention Science, University of Basel, 4001 Basel, Switzerland; 31 Department of Psychology, University of Cyprus, 1678 Nicosia, Cyprus; 32 Department of Nursing, Cyprus University of Technology, 3036 Limassol, Cyprus

**Keywords:** Prosociality, Coronavirus, Adherence, Disease containment measures, Longitudinal study

## Abstract

**Background:**

Identifying common factors that affect public adherence to COVID-19 containment measures can directly inform the development of official public health communication strategies. The present international longitudinal study aimed to examine whether prosociality, together with other theoretically derived motivating factors (self-efficacy, perceived susceptibility and severity of COVID-19, perceived social support) predict the change in adherence to COVID-19 containment strategies.

**Method:**

In wave 1 of data collection, adults from eight geographical regions completed online surveys beginning in April 2020, and wave 2 began in June and ended in September 2020. Hypothesized predictors included prosociality, self-efficacy in following COVID-19 containment measures, perceived susceptibility to COVID-19, perceived severity of COVID-19 and perceived social support. Baseline covariates included age, sex, history of COVID-19 infection and geographical regions. Participants who reported adhering to specific containment measures, including physical distancing, avoidance of non-essential travel and hand hygiene, were classified as adherence. The dependent variable was the category of adherence, which was constructed based on changes in adherence across the survey period and included four categories: non-adherence, less adherence, greater adherence and sustained adherence (which was designated as the reference category).

**Results:**

In total, 2189 adult participants (82% female, 57.2% aged 31–59 years) from East Asia (217 [9.7%]), West Asia (246 [11.2%]), North and South America (131 [6.0%]), Northern Europe (600 [27.4%]), Western Europe (322 [14.7%]), Southern Europe (433 [19.8%]), Eastern Europe (148 [6.8%]) and other regions (96 [4.4%]) were analyzed. Adjusted multinomial logistic regression analyses showed that prosociality, self-efficacy, perceived susceptibility and severity of COVID-19 were significant factors affecting adherence. Participants with greater self-efficacy at wave 1 were less likely to become non-adherence at wave 2 by 26% (adjusted odds ratio [aOR], 0.74; 95% CI, 0.71 to 0.77; *P* < .001), while those with greater prosociality at wave 1 were less likely to become less adherence at wave 2 by 23% (aOR, 0.77; 95% CI, 0.75 to 0.79; *P* = .04).

**Conclusions:**

This study provides evidence that in addition to emphasizing the potential severity of COVID-19 and the potential susceptibility to contact with the virus, fostering self-efficacy in following containment strategies and prosociality appears to be a viable public health education or communication strategy to combat COVID-19.

**Supplementary Information:**

The online version contains supplementary material available at 10.1186/s12992-023-00928-7.

## Introduction

Since the outbreak of coronavirus disease (COVID-19) in 2019, government leaders worldwide have used various measures to contain its spread, such as physical distancing, avoiding large gatherings, wearing masks and frequent hand washing [1]. Research has been conducted to assess the impact of these containment measures [2]. For instance, cancelling small gatherings has been found to decrease the effective reproduction number of severe acute respiratory syndrome coronavirus 2 (SARS-CoV-2) (R_t_) by up to 83% [3]; while self-isolation, household quarantine and manual contact tracing can reduce COVID-19 transmission up to 64% [4].

Public adherence to disease containment measures remains crucial for controlling the spread of COVID-19. However, such adherence varies depending on the types of measures required to be complied with, the intensities of governmental enforcement measures and is influenced by the interplay of demographic, political, and sociocultural factors [5–7]. In a longitudinal study conducted between March and December 2020 involving 238,797 participants from 14 countries, adherence to lower-cost and habituating behaviors (e.g., mask-wearing in crowded areas) was found to increase progressively over time [8], while adherence to high-cost, sensitizing behaviors (e.g., physical distancing, avoiding crowds) gradually decreased due to pandemic fatigue (i.e., overall tiredness or demotivation to follow recommended protective behaviors) and reduced risk perception of COVID-19 [8].

Psychological factors that influence adherence to COVID-19 related protective behaviors have been recently examined in several theoretical frameworks [6]. The Health Belief Model [9], the Social Cognitive Theory[10], the Reasoned Action Approach [11] and the Health Action Process Approach are theories centered around self-motivation that have been increasingly adopted in COVID-19-related research [12, 13]. These theories suggest that increased self-efficacy, stronger perceived susceptibility to COVID-19 infection and better treatment outcome expectancies are associated with increased adherence to disease containment measures [6], while lack of social support is identified as a behavioral barrier to adherence [13]. The Capability, Opportunity and Motivation Behavior (COM-B) model is another theoretical model that has been used in recent literature related to COVID-19 [14–16], suggesting that changing an individual’s behavior to combat COVID-19 can be affected by (1) capabilities, which are the relevant knowledge and skills to engage in that particular behavior; (2) opportunities, referring to resources, cultural norms and/or social support and cues to facilitate the execution of the behavior; and (3) motivation to drive behavioral change [15]. In our previous international surveys, we adopted the Leventhal’s Common-Sense Model of Self-Regulation and found that risk perceptions of COVID-19 could shape adherence to containment measures, mediated by less avoidance-based coping and better self-efficacy in disease prevention and management [17, 18].

The literature has undergone extensive expansion in recent years regarding the investigation of factors that determine adherence to COVID-19 containment measures. A review of 29 studies conducted in western countries suggests that people who are female, older, have higher socioeconomic status, trust in government or health authorities, trust in science or medicine and access information from traditional media sources are more likely to adhere to COVID-19 related containment measures [19]. This may be partly due to the increased concern about their own health risks held among these groups [5, 7, 20, 21]. Fear and perceived personal threat of COVID-19 can also improve adherence [22], but both could also lead to anxiety and avoidance behaviors that may lead to lower adherence [23, 24]. Cultural norms and practices (e.g., cultures that prioritize collectivism over individualism may be more likely to adhere to measures that benefit the group as a whole) [25], communication-related factors (e.g., clarity, effectiveness, reach of communication campaigns, channels and methods used to disseminate relevant COVID-19 information) may also shape attitudes and behaviors in response to various containment measures [26].

In view of the communal nature of the COVID-19 pandemic, there has been a call to explore the role of prosociality in the context of curbing the spread of COVID-19. Prosociality refers to an attitude or a set of voluntary actions that an individual may adopt to help, care for, or comfort others [27]. As suggested by the Social Identity Theory [28], the sense of belonging, identity and prosociality would often increase within a group in supporting one another if group members perceive themselves as facing crises [29]. Prosociality requires people to think and act collectively with kindness, cooperation and sensitivity to others’ welfare (e.g., protect others from COVID-19). It has recently been considered as an important target of public health interventions to promote adherence to disease containment measures [30–33], adopting mobile applications for contact tracing [34] and vaccine uptake [35, 36]. However, the association between prosociality and adherence or uptake of COVID-19-related measures has only been demonstrated in single-center, cross-sectional and correlational studies [32–34, 36, 37], in which changing patterns of adherence behaviors have been neglected. Indeed, variations in moral obligations, cultural values, social norms and public leaderships across regions may affect the degree to which individuals prioritize the well-being of others over their own self-interests [38, 39]. Furthermore, differences in the severity and prevalence of COVID-19 across regions may impact individuals' perceived needs for adherence to related containment measures. Therefore, in this longitudinal study involving an international sample of participants, we aimed to examine whether prosociality, along with other known self-focused motivating factors such as self-efficacy, perceived susceptibility and severity of COVID-19, and perceived social support, can predict changes in adherence to COVID-19 containment measures during the first year of the pandemic. This study will also take into account other demographic factors and the status of COVID-19.

## Methods

### Design and study participants

The study was part of the international COVID-IMPACT survey, which aimed to examine the psychological and behavioral responses of the public to COVID-19 and its related containment measures [18, 30, 40, 41]. Between April and June 2020, the first wave of the survey (wave 1) was administered to a sample of adults aged 18 years or older who were able to read at least one of the following languages: English, Chinese, Spanish, French, German, Turkish, Portuguese, Italian, Dutch, Polish, Romanian, Greek, Hungarian, Persian, Finnish, Slovenian, Latvian, and Montenegrin. These individuals were recruited from 51 countries across the globe through various means, such as press outlets (e.g., newspapers, newsletters, radio stations), social media platforms, professional groups' mailing lists, and networks, as well as mass mailings from participating universities. No exclusion criteria for participation were set. Those who volunteered to participate in the study were invited to provide their informed consent electronically and complete a 20-min survey on a secure online platform. Upon completion, the participants were directed to another secured platform to indicate whether they would like to be contacted for follow-up data collection and to provide their email addresses. If agreed, they were recontacted between August and September 2020 for the second wave of the survey (wave 2). Since the time spent to administer each survey was approximately less than 20 min, no compensation was provided. The study received ethics approval from the Cyprus National Bioethics Committee (ref.: EEBK E* 2020.01.60) and by the local ethics boards whose research team members were involved in collecting data. Our reporting followed the Strengthening the Reporting of Observational Studies in Epidemiology (STROBE) reporting guidelines [42].

### Measures

#### Adherence to COVID-19 containment measures

In both wave 1 and wave 2 of the survey, participants were asked to rate their level of adherence to (1) physical distancing, (2) self-isolation, avoidance of non-essential travel, and (3) hand hygiene during the pandemic, with response options ranging from '1' for non-adherence to '10' for fully adherence. Participants who responded '7 = mostly adherent’ or higher for all three items were considered as adherence. The following explanatory variables were assessed at wave 1:

#### Self-efficacy

Five items (response: ‘1’ = strongly disagree to ‘7’ = strongly agree) based on Bandura's self-efficacy theory were adopted to measure whether the participants perceived themselves as competent in following the COVID-19 containment measures. An example item is “I have the skills to get through this difficult situation”, with a higher total score indicating better self-efficacy [17].

#### Perceived susceptibility and severity

Six items (response: ‘1’ = strongly disagree to ‘6’ = strongly agree) based on the Health Belief Model were used to assess the participants’ overall illness perceptions toward COVID-19, with higher total scores indicating stronger perceptions. One example item assessing perceived susceptibility is “I am concerned about the risk of getting the COVID-19”, while an example item assessing perceived severity was “My life will change if I get infected by COVID-19” [43].

#### Prosociality

Six items (response: ‘1’ = never to ‘5’ = always) adapted from the Prosocialness Scale for Adults were used to assess six different prosocial behaviors during the COVID-19 pandemic, such as sharing, helping, taking care of others and showing empathy. Example items are ‘I try to help others’ and ‘I am empathic with those in need’. A higher total score indicates greater prosocial motivation [44].

#### Perceived social support

Three items of the Oslo Social Support Scale were used to assess the availability of social support by asking the number of people that were closed to the participant (response: ‘1’ = none to ‘4’ = more than 5 people), the extent of interest and concern that people show in what the participants did (response: ‘1’ = none to ‘5’ = a lot) and the possibility of getting practical help from neighbors (response: ‘1’ = very difficult to ‘5’ = very easy). A higher total score indicates better social support [45].

The psychometric properties of the aforementioned measures have been stated in our previous publications [18, 30, 40, 41], with acceptable internal consistencies across study regions (Cronbach’s alphas = 0.76 to 0.85) and satisfactory construct validity (*r*s = 0.68 to 0.82) to their corresponding validation measures [18, 30, 40, 41]. Details of the measures appear in Supplementary Table 1.

#### Sociodemographic variables

The following sociodemographic variables were assessed at wave 1, including sex (male/ female), age in years (18–30/ 31–59/ ≥ 60), educational level (higher school or below/ college or university/ postgraduate or above/ others), marital status (single/ in a relationship or engaged/ married/ others), having children (yes/no), employment status (working as full-time/ part-time/ unemployed/ parental leave/ retired), working as a healthcare professional (yes/no), as well as the personal, partner and the significant others’ history of COVID-19 infection (yes/ unsure/ no). Geographical region was also included, following the recommended classifications given by the Population Division of the Department of Economic and Social Affairs of the United Nation (East Asia/ West Asia/ North and South America/ Northern Europe/ Western Europe/ Southern Europe/ Eastern Europe or others) [46]. Details of the countries involved in these regions appear in Supplementary Table 2.

### Statistical analysis

To assess changes of adherence to COVID-19 containment measures in the two waves of surveys, the participants were classified into four types of adherence groups, including (1) the ‘non-adherence group’, which referred to those who scored less than seven on all three question items assessing adherence to COVID-19 containment measures in both waves of surveys; (2) the ‘less adherence group’, which referred to those who showed a reduced number of question items with scores equal to or larger than seven in wave 2 when compared to wave 1; (3) the ‘sustained adherence group’, referring to those reported scores greater than seven on all three question item in both waves of surveys; and (4) the ‘greater adherence group’, referring to those who had an increase number of question items with scores equal to or greater than seven in wave 2 when compared to wave 1. Subsequently, descriptive data on sociodemographic characteristics of the participants and outcome measures were computed and summarized. Univariate analyses, such as one-way analysis of variance (ANOVA) and chi-square test, were conducted to examine whether the means or distributions of proportions of explanatory variables differed significantly across the four adherence groups. Multinomial logistic regression was conducted to examine whether those plausible factors, including prosociality, self-efficacy, perceived susceptibility and severity of COVID-19 and perceived social support, were associated with the change in adherence to COVID-19 containment measures. This analysis was adjusted for covariates (region, sex, age and history of COVID-19 infection) that showed significant differences in the distributions of proportions of their corresponding attributes across the four adherence groups. Results of regression analyses were reported as odds ratio (OR) with 95% confidence interval (CI). All statistical tests conducted in IBM SPSS 26.0 (IBM Corp, Armonk: NY, USA) were considered significant at *P-*value < 0.05, two-sided.

## Results

### Sample characteristics

Among the 9565 participants who completed wave 1 of the survey, 8948 participants (93.5%) agreed to be recontacted. Of these, 2189 (24.4%) were successfully re-contacted to provide the follow-up data. The participants (82% female, 57.2% aged 31–59 years) were from East Asia (217 [9.7%]), West Asia (246 [11.2%]), North and South America (131 [6.0%]), Northern Europe (600 [27.4%]), Western Europe (322 [14.7%]), Southern Europe (433 [19.8%]), Eastern Europe (148 [6.8%]) and other regions (96 [4.4%]). The participants mainly had a postgraduate degree (1058 [48.3%]), were married (821 [37.5%]), had children (902 [58.8%]), worked full-time (1235 [56.4%] and less than one-fifth (374 [17.4]) were health care professionals. Almost half of the participants (1206 [55.1%]) sustained adherence in both waves of surveys. We conducted wave 1 of the survey in April 2020 and therefore less than one-tenth of respondents (28 [1.3%]), their partners (24 [1.1%]) and significant others (136 [6.2%]) were infected with COVID-19. Compared to the other three adherence groups, the sustained adherence group (1206 [55.1%]) reported the highest scores in the self-efficacy of following the COVID-19 containment measures (*M* = 26.21), perceived susceptibility (*M* = 9.14) and perceived severity of COVID-19 (*M* = 13.21), as well as prosociality (*M* = 22.87), respectively (see Table 1).

**Table 1:** Characteristics of the participants by level of adherence to COVID-19 containment measures

Characteristics	All (*N* = 2189)	Non-adherence group (*n* = 219)	Less adherence group (*n* = 674)	Sustained adherence group (*n* = 1206)	Greater adherence group (*n* = 90)	χ^2^ *or F(df*)/	*P* value
Region^a^, No. (%)							
East Asia	213 (9.7)	21 (9.6)	27 (4.0)	148 (12.3)	17 (18.9)	145 (21)	< .001
West Asia	246 (11.2)	32 (14.6)	81 (12.0)	128 (10.6)	5 (5.6)		
North and South America	131 (6.0)	7 (3.2)	20 (3.0)	93 (7.7)	11 (12.2)		
Northern Europe	600 (27.4)	44 (20.1)	179 (26.6)	356 (29.5)	21 (23.3)		
Western Europe	322 (14.7)	47 (21.5)	135 (20.0)	123 (10.2)	17 (18.9)		
Southern Europe	433 (19.8)	36 (16.4)	131 (19.4)	253 (21.0)	13 (14.4)		
Eastern Europe	148 (6.8)	22 (10.0)	69 (10.2)	56 (4.6)	1 (1.1)		
Others	96 (4.4)	10 (4.6)	32 (4.7)	49 (4.1)	5 (5.6)		
Sex, No. (%)							
Male	394 (18.0)	70 (32.0)	108 (16.0)	191 (15.8)	25 (27.8)	40.37 (3)	< .001
Female	1795 (82.0)	149 (68.0)	566 (84.0)	1015 (84.2)	65 (72.2)		
Age in years, No. (%)							
Young adults (18–30 years)	782 (35.7)	99 (45.2)	243 (36.1)	407 (33.7)	33 (36.7)	17.02 (6)	.009
Middle-aged (31–59 years)	1253 (57.2)	111 (50.7)	394 (58.5)	699 (58.0)	49 (54.4)		
Older adults (≥ 60 years)	154 (7.0)	9 (4.1)	37 (5.5)	100 (8.3)	8 (8.9)		
Educational level, No. (%)							
Higher school or below	212 (9.7)	30 (13.7)	68 (10.1)	107 (8.9)	7 (7.8)	10.77 (9)	.29
College or university	884 (40.4)	92 (42.0)	262 (38.9)	495 (41.9)	35 (38.9)		
Postgraduate or above	1058 (48.3)	91 (41.6)	332 (49.3)	588 (48.8)	47 (52.2)		
Others	35 (1.6)	6 (2.7)	12 (1.8)	16 (1.3)	1 (1.1)		
Marital status, No. (%)							
Single	657 (30.0)	83 (37.9)	192 (28.5)	354 (29.4)	28 (31.1)	10.44 (9)	.32
In a relationship/ engaged	556 (25.4)	57 (26.0)	169 (25.1)	307 (25.5)	23 (25.6)		
Married	821 (37.5)	67 (30.6)	259 (38.4)	463 (38.4)	32 (35.6)		
Others (divorced/widowed/separated)	155 (7.1)	12 (5.5)	54 (8.0)	82 (6.8)	7 (7.8)		
Having children, No. (%)							
Yes	902 (41.2)	74 (33.8)	274 (40.7)	516 (42.8)	38 (42.2)	6.34 (3)	.10
No	1287 (58.8)	145 (66.2)	400 (59.3)	690 (57.2)	52 (57.8)		
Employment status, No. (%)							
Full-time	1235 (56.4)	127 (58.0)	374 (55.5)	683 (56.6)	51 (56.6)	16.99 (12)	.15
Part-time	363 (16.6)	29 (13.2)	123 (18.2)	196 (16.3)	15 (16.7)		
Unemployed	442 (20.2)	50 (22.8)	133 (19.7)	237 (19.7)	22 (24.4)		
Parental leave	50 (2.3)	9 (4.1)	16 (2.4)	25 (2.1)	0 (0.0)		
Retired	99 (4.5)	4 (1.8)	28 (4.2)	65 (5.4)	2 (2.2)		
Working as health care professionals^b^, No. (%)							
Yes	374 (17.4)	39 (17.9)	125 (18.9)	193 (16.3)	17 (19.1)	2.25 (3)	.52
No	1780 (82.6)	179 (82.1)	537 (81.1)	992 (83.7)	72 (80.9)		
Have you been infected by COVID-19?, No. (%)							
Yes	28 (1.3)	7 (3.2)	9 (1.3)	11 (0.9)	1 (1.1)	32.29 (6)	< .001
I am not sure but I have symptoms	234 (10.7)	40 (18.3)	85 (12.6)	99 (8.2)	10 (11.1)		
No	1927 (88.0)	172 (78.5)	580 (86.1)	1096 (90.9)	79 (87.8)		
Have your partner been infected by COVID-19?, No. (%)						9.53 (6)	.15
Yes	24 (1.1)	4 (1.9)	6 (0.9)	13 (1.1)	1 (1.1)		
I am not sure but I have symptoms	149 (6.9)	21 (9.7)	55 (8.2)	70 (5.9)	3 (3.4)		
No	1990 (92.0)	191 (88.4)	608 (90.9)	1107 (93.0)	84 (95.5)		
Has your significant others being infected by COVID-19, No. (%)						6.46 (6)	.37
Yes	136 (6.2)	12 (5.5)	46 (6.8)	70 (5.8)	8 (8.9)		
I am not sure but I have symptoms	196 (9.0)	24 (11.0)	69 (10.2)	95 (7.9)	8 (8.9)		
No	1857 (84.8)	183 (83.6)	559 (82.9)	1041 (86.3)	74 (82.2)		
Self-efficacy, mean (SD)	25.69 (3.69)	23.67 (4.89)	25.56 (3.64)	26.21 (3.26)	24.58 (4.11)	34.50 (3)	< .001
Perceived susceptibility, mean (SD)	8.75 (3.45)	7.83 (3.57)	8.40 (3.36)	9.14 (3.43)	8.50 (3.39)	13.03 (3)	< .001
Perceived severity, mean (SD)	12.63 (3.71)	10.98 (3.97)	12.07 (3.66)	13.21 (3.56)	13.07 (3.51)	30.97 (3)	< .001
Prosociality, mean (SD)	22.66 (4.08)	21.87 (4.27)	22.66 (3.99)	22.87 (4.07)	22.00 (4.14)	4.63 (3)	.004
Perceived social support, mean (SD)	9.81 (2.12)	9.50 (2.19)	9.99 (2.08)	9.79 (2.11)	9.53 (2.33)	3.83 (3)	.012

^a^In this study, East Asia included Hong Kong; West Asia included Cyprus and Turkey; Northern and Southern America included Colombia and the United States; Northern Europe included The United Kingdom, Finland, Ireland and Latvia; Western Europe included Switzerland, Germany, Austria and France; Southern Europe included Greece, Spain, Italy, Portugal and Montenegro; Eastern Europe included Poland, Romania and Hungary

^b^1.6% missing data

### Predictors of changing patterns of adherence to COVID-19 containment measures

The multinomial logistic regression model was statistically significant (χ2 (51) = 379.41, *P* < .001), with a total variance of 28.1% explained (Nagelkerke *R*^2^) and correctly classifying 69.5% of cases. Two sociodemographic covariates, which were sex and history of COVID-19 infection, were predictive of adherence. Taking the ‘sustained adherence group’ as a reference group, male (adjusted odds ratio [aOR], 2.34; 95% CI 1.64 to 3.35; *P* < .001), those who were infected with COVID-19 or had COVID-19 symptoms at wave 1 were more likely to become non-adherence at wave 2 (aOR, 2.37, 95% CI 1.55 to 3.63; *P* < .001). Self-efficacy (aOR = 0.74, 95% CI 0.71 to 0.77; *P* < .001), perceived susceptibility (aOR = 0.93, 95% CI 0.88 to 0.99; *P* = 0.02), perceived severity (aOR = 0.90, 95% CI 0.85 to 0.94; *P* < .001), and prosociality (aOR = 0.85, 95% CI 0.81 to 0.89; *P* = .008) measured at wave 1 were significant factors in lowering the risk of becoming non-adherence at wave 2. Similarly, self-efficacy (aOR = 0.93, 95% CI 0.91 to 0.96; *P* < .001), perceived susceptibility (aOR = 0.96, 95% CI 0.93 to 1.00; *P* = .04), perceived severity (aOR = 0.96, 95% CI 0.93 to 0.99; *P* = .02), and prosociality (aOR = 0.77, 95% CI 0.75 to 0.79; *P* = .04) measured at wave 1 were significant factors in lowering the risk of becoming less adherence at wave 2. Notably, attaining better self-efficacy at wave 1 reduced the likelihood of being non-adherence at wave 2 by 26% (aOR = 0.74), while being more prosocial at wave 1 reduced the likelihood of becoming less adherence at wave 2 by 23% (aOR = 0.77, see Table 2).

**Table 2:** Results of the multinomial logistic regression investigating predictors of changing patterns of adherence of COVID-19 containment measures

	Non-adherence group (*N* = 219)			Less adherence group (*N* = 674)			Greater adherence group (*N* = 90)		
	aOR	95% CI	*P* value	aOR	95% CI	*P* value	aOR	95% CI	*P* value
Region^a^									
East Asia	1.05	0.42–2.63	.91	0.32	0.17–0.60	< .001	1.18	0.39–3.52	.77
West Asia	1.96	0.82–4.73	.13	1.11	0.65–1.92	.70	0.53	0.14–1.08	.34
North and South America	0.58	0.19–1.76	.34	0.39	0.20–0.76	.005	1.39	0.45–4.35	.58
Northern Europe	0.84	0.37–1.93	.69	0.81	0.50–1.33	.41	0.64	0.22–1.81	.40
Western Europe	2.34	1.01–5.42	.05	1.66	0.99–2.78	.06	1.47	0.50–4.30	.48
Southern Europe	1.38	0.59–3.21	.46	0.92	0.56–1.53	.75	0.62	0.21–1.85	.39
Eastern Europe	2.44	0.97–6.16	.06	1.98	1.11–3.54	.02	0.21	0.02–1.92	.17
Others	[Reference]			[Reference]			[Reference]		
Sex									
Male	2.34	1.64–3.35	< .001	1.02	0.77–1.34	.92	1.84	0.98–3.08	.05
Female	[Reference]			[Reference]			[Reference]		
Age in years									
Young adults (18–30 years)	1.44	0.67–3.13	.35	1.30	0.83–2.01	.25	.746	0.31–1.78	.51
Middle-aged (31–59 years)	1.02	0.48–2.17	.96	1.21	0.80–1.84	.36	.745	0.33–1.69	.48
Older adults (≥ 60 years)	[Reference]			[Reference]			[Reference]		
Infected by COVID-19									
Yes/ have symptoms	2.37	1.55–3.63	< .001	1.35	0.99–1.84	.06	1.31	0.66–2.60	.44
No symptoms	[Reference]			[Reference]			[Reference]		
Motivating factors									
Self-efficacy	0.74	0.71–0.77	< .001	0.93	0.91–0.96	< .001	0.88	0.84–1.05	.05
Perceived susceptibility	0.93	0.88–0.99	.02	0.96	0.93–1.00	.04	0.94	0.87–1.01	.10
Perceived severity	0.90	0.85–0.94	< .001	0.96	0.93–0.99	.02	1.00	0.92–1.07	.90
Prosociality	0.85	0.81–0.89	.008	0.77	0.75–0.79	.04	0.97	0.92–1.03	.31
Perceived social support	1.00	0.93–1.09	.95	1.028	0.98–1.08	.32	1.01	0.90–1.14	.82

^a^In this study, East Asia included Hong Kong; West Asia included Cyprus and Turkey; Northern and Southern America included Colombia and the United States; Northern Europe included The United Kingdom, Finland, Ireland and Latvia; Western Europe included Switzerland, Germany, Austria and France; Southern Europe included Greece, Spain, Italy, Portugal and Montenegro; Eastern Europe included Poland, Romania and Hungary

Reference category for the dependent variable is the sustained adherence group (*N* = 1206)

## Discussion

The current study investigated the changes of adherence in COVID-19 containment measures in a large convenience sample of adults from eight geographical regions between April and September 2020 in which the early stage of COVID-19 pandemic occurred. Half of the respondents fully adhered to the suggested strategies, such as physical distancing, self-isolation, and hand hygiene, at six months follow-up after the baseline. Across all the groups in accordance with the patterns of adherence behaviors, the odds of not adhering to all strategies increased at least twice if the participants were male and infected with COVID-19. Indeed, the differences in sex and COVID-19 status affecting adherence to COVID-19 containment measures have been discussed in the literature [47–50], in which lower levels of perceived threats of illness and adherence among males are considered as important factors explaining the increased rates of COVID-19 related morbidity and mortality compared to women [47–50]. Our findings are also consistent with the existing body of knowledge supporting that various sociodemographic factors, such as older age [50], caregivers of elderly or children and working as part-time or retired, could affect adherence [5, 51].

Self-efficacy, perceived susceptibility and severity of COVID-19 were found to be the significant factors in lowering the risks of becoming non-adherence and less adherence to COVID-19 containment measures at follow-up. Interestingly, we found that self-efficacy attained a larger reduction in the risk of being non-adherent than that of perceived susceptibility and severity, suggesting that an individual’s belief that one can carry out and adhere to COVID-19 containment measures is more important than one’s beliefs about the severity of COVID-19 or the risk of getting infected [52]. Indeed, the Health Action Process Approach also indicates that self-efficacy is the proximal determinant for developing an intention to change behavior, while risk perception is considered a distal determinant [12].

Our adjusted analysis showed that prosociality contributed significant reductions in the risks of becoming non-adherence and less adherence to COVID-19 containment measures at follow-up (i.e., 15% and 23%, respectively). COVID-19 containment measures (e.g., vaccinations and social distancing) have been recently discussed as global public good where people can 'free-ride' on others: receiving social benefits from others (e.g., reduced risk of infection as others follow the rules) without paying for the costs (e.g., continue dining out) [53]. Hence, promoting adherence to COVID-19 containment measures has been recently framed as a prosocial act. The extant literature has identified a number of contributors that shape the prosocial motivations in the face of collective action problems (e.g., public health crises like COVID-19), including personality traits, individual values, core political values, empathy and sympathy toward individuals vulnerable to the problem [22, 54]. Our findings further extend the understanding of prosociality, in which its protective role on non-adherence still remains and even exerts a stronger, longitudinal effect when simultaneously compared to that of self-efficacy in spite of the heterogeneity of the participants’ characteristics across study regions.

We found that perceived social support did not affect the patterns of adherence to the COVID-19 containment measures. This finding is contrast with those of recent studies indicating that individuals who perceive more social support from their friends can influence their health behaviors either positively or negatively, due to social norms or peer pressure [55, 56]. Of note, as a result of social distancing measures (e.g., lockdowns, work-from-home arrangements) implemented during the survey period, it was more difficult for individuals to interact socially and/or gather, which explained the relatively low perceived level of social support.

### Study strengths and limitations

Our findings were based on an international, geographically diverse sample and the timing of data collection was within one month after the World Health Organization declared COVID-19 as a pandemic. In addition, we classified the participants into subgroups in accordance with their different patterns of adherence. The understanding of the predictors and covariates in each subgroup provides future directions for tailoring public health programs or messages in promoting adherence to COVID-19 containment measures.

It is important to consider the limitations of our study when interpreting the results. Our study was conducted in two phases, and the assumption of linearity of associations between variables limits the scope of a more advanced longitudinal analysis, which considers time effect as an additional covariate. In order to better examine the effect of changes in health-related perception variables on adherence behaviors over time, future research could employ latent growth curve modelling analysis to evaluate the trajectory of change in both independent and dependent variables. This analytical approach allows for the modeling of within-person and between-person variability in the data, thus providing a more comprehensive understanding of the relationship between the variables over time [57, 58]. While the instruments used in our study were found to have satisfactory psychometric properties in terms of validity and reliability, we did not conduct a multiple-group factor analysis alignment to further examine the measurement equivalence or invariance of these instruments across different cultural or linguistic groups [59]. Implementing this method could have strengthened the cross-cultural validity of our study and ensured that our findings could be applied to a broader population.

Our assessment of adherence to COVID-19 containment measures deserves attention. The first wave of our survey was conducted in April 2020, during the early stage of the pandemic when there was a lack of consensus among global political and public health leaders on the necessity of wearing masks in community settings [60, 61]. Misinformation regarding the utility of masks, potential adverse effects, and severe shortages of medical resources, including medical masks, further complicated the situation [60, 61]. As a result, universal adoption of mask wearing was not achieved [62], and we did not collect data on participants' adherence to this measure over time. For future studies investigating adherence patterns to various containment measures during outbreaks of infectious diseases, assessing adherence to proper face mask wearing is highly recommended to gain a more comprehensive understanding of adherence behaviors. As the assessments were based on self-reports, which were regarded as the best capture of how people early responded to the rapid-changing nature of COVID-19. Nevertheless, objective measurement of adherence to health behaviors are often preferable and less susceptible to response biases. The use of tracking technologies such as mobile apps, wearable devices, or location tracking tools may also potentially provide a more accurate understanding of real-time adherence behaviors [63].

It is noteworthy that the regression analysis included key sociodemographic and health-related perception variables based on Bandura's self-efficacy theory, the Social Cognitive Theory, and the Health Belief Model, which achieved a correct classification rate of up to 69.5%. However, other contextual or social determinants of adherence to containment measures could be considered in future research, such as place of living, housing quality, political polarization and inclinations, trust in government and scientific evidence, susceptibility to misinformation, as well as health or e-health literacy levels [64, 65]. Additionally, regional differences in adherence to COVID-19 containment measures may be attributed to a variety of factors, cultural disparities, access to healthcare and information, socioeconomic status, and government policies, as well as initial perceptions and response to COVID-19. However, in view of the small sample size in some regions (e.g., East Asia, Eastern Europe and other regions, each was less than 10% of the total samples), subgroup analysis was not feasible in our study. While we adjust for geographic region in our regression analysis to account for potential regional differences in adherence, the aforementioned factors warrant further investigation in future studies. Finally, over 80% of the participants were female and the survey was conducted via online which may limit the generalizability of our findings to other populations. Further research with more diverse samples is needed to confirm our results and to better understand the impact of personal factors on adherence to COVID-19 and other related infection prevention measures.

## Conclusions

Our investigations remind that across regions and cultures, some inner qualities of a human being, such as acting prosocially through helping and supporting for the benefit of others and perceived competence in executing behaviors can affect individuals in taking small steps to contain the COVID-19 spread. Pandemic-specific public health communication and behavioral intervention efforts should possess qualities of prosociality as a powerful altruistic motivator for better adherence to COVID-19 containment measures, alongside with the support of scientific evidence, social norm and consensus which increase an individual’s self-efficacy [66, 67]. Deontological messages highlighting the importance of societal and communal benefits (e.g., protect others) rather than the benefit to oneself (e.g., protect yourself) in times of public health crisis may be especially effective for people to engage in health policy-relevant behaviors.

## Supplementary Information

**Additional file 1: Supplementary Table 1.** Measures used in the COVID-IMPACT study.

**Additional file 2: Supplementary Table 2.** Number of the participants involved in the study from each country and geographical region.

## Data Availability

The datasets used and/or analysed during the current study are available from the corresponding author on reasonable request.

## Abbreviations

ANOVA: Analysis of variance
aOR: Adjusted odds ratio
COVID-19: Coronavirus disease 2019
MD: Mean difference
