# Supplementary material for: Predictors of changing patterns of adherence to containment measures during the early stage of COVID-19 pandemic: an international longitudinal study
Source: Global Health. 2023 Apr 17;19:25. doi: 10.1186/s12992-023-00928-7 (PMC10106884; doi:10.1186/s12992-023-00928-7)
Supplement: Supplementary file 1 — Additional file 1: Supplementary Table 1. Measures used in the COVID-IMPACT study. [file 12992_2023_928_MOESM1_ESM.docx]

**Supplementary Table 1.** Measures used in the COVID-IMPACT study

| **Adherence to COVID-19 containment measures** |
| --- |
| Instruction: Please select which of the following measures you are currently adhering and to which extend |
| 1 = Non-adherent  2-3 = Somewhat non-adherent  4-6 = Moderate adherence  7-8 = Mostly adherent  9-10 = Fully adherent |
| 1. Keeping distance from other people when going out  1 2 3 4 5 6 7 8 9 10  2. Self-isolating and avoidance of non-essential travel according to national guidelines  1 2 3 4 5 6 7 8 9 10  3. Washing hands regularly with water and soap  1 2 3 4 5 6 7 8 9 10 |

| **Self-Efficacy** |
| --- |
| Instruction: Please indicate how much you agree with the statements below in terms of your capability to follow recommendations for social distancing (staying home as much as possible). |
| 1= strongly disagree, 7 = strongly agree |
| 1. I have the skills to get through this difficult situation.   1 2 3 4 5 6 7   1. I can deal with this difficult situation.   1 2 3 4 5 6 7   1. When facing difficulties in following the recommendations, I am certain that I will overcome them.   1 2 3 4 5 6 7   1. Compared to other people, I can follow these recommendations pretty well.   1 2 3 4 5 6 7   1. Even when things get tough, I can follow these recommendations quite well.   1 2 3 4 5 6 7 |

| **Perceived Susceptibility and Severity** |
| --- |
| Instruction: Please indicate how much you agree with the statements below in terms of your perceptions toward COVID-19 infection. |
| \| \| 1=STRONGLY DISAGREE **(SD)** 2=MODERATELY DISAGREE **(MD)** 3=SLIGHTLY DISAGREE **(D)** \| 4=SLIGHTLY AGREE **(A)** 5=MODERATELY AGREE **(MA)** 6=STRONGLY AGREE **(SA)** \| \| --- \| --- \| \| \| --- \| --- \| --- \| |
| \|  \| \| **SD** \| **MD** \| **D** \| **A** \| **MA** \| **SA** \| \| --- \| --- \| --- \| --- \| --- \| --- \| --- \| --- \| \| 1 \| I have an increased risk of getting infected by COVID-19 \| 1 \| 2 \| 3 \| 4 \| 5 \| 6 \| \| 2 \| I am concerned about the risk of getting the COVID-19 \| 1 \| 2 \| 3 \| 4 \| 5 \| 6 \| \| 3 \| I get sick more easily than other people my age \| 1 \| 2 \| 3 \| 4 \| 5 \| 6 \| \| 4 \| COVID-19 may lead to serious health problems \| 1 \| 2 \| 3 \| 4 \| 5 \| 6 \| \| 5 \| I am afraid that if I get infected by COVID-19 I will be very sick \| 1 \| 2 \| 3 \| 4 \| 5 \| 6 \| \| 6 \| My life will change if I get infected by COVID-19 \| 1 \| 2 \| 3 \| 4 \| 5 \| 6 \|   [items 1-3: perceived susceptibility, items 4-6 perceived severity |

| **Prosociality** |
| --- |
| Instruction: The following statements describe a large number of common situations. There are no right or wrong answers; the best answer is the immediate, spontaneous one. Read each phrase carefully and fill in the number that reflects your first reaction. |
| 1 = Never  2 = Rarely  3 = Occasionally  4 = Often  5 = Always |
| 1. I am pleased to help my friends/colleagues in their activities.  2. I share the things that I have with my friends.  3. I try to help others.  4. I am available for volunteer activities to help those who are in need.  5. I am empathic with those who are in need.  6. I spend time with those friends who feel lonely. |

| **Perceived Social Support (Oslo Social Support, OSS-3)** |
| --- |
| Instruction: This questionnaire asks about the social support you receive from others. Social support means the help and assistance you receive from other people, such as family, friends, neighbors, and coworkers. There are no right or wrong answers, so please respond to each statement based on your own experiences. Please read each statement carefully and select the response that best reflects your feelings in the last month. |
| 1. How many people are so close to you that you can count on them if you have great personal problems?   1 = ‘none’  2 = ‘1–2’  3 = ‘3–5’  4 = ‘5+’     1. How much interest and concern do people show in what you do?   1 = ‘none’  2 = ‘little’  3 = ‘uncertain’  4 = ‘some’  5 = ‘a lot’     1. How easy is it to get practical help from neighbors if you should need it?   1 = ‘very difficult’  2 = ‘difficult’  3 = ‘possible’  4 = ‘easy’  5 = ‘very easy’ |
