# Supplementary material for: Predictors of changing patterns of adherence to containment measures during the early stage of COVID-19 pandemic: an international longitudinal study
Source: Global Health. 2023 Apr 17;19:25. doi: 10.1186/s12992-023-00928-7 (PMC10106884; doi:10.1186/s12992-023-00928-7)
Supplement: Supplementary file 2 — Additional file 2: Supplementary Table 2. Number of the participants involved in the study from each country and geographical region. [file 12992_2023_928_MOESM2_ESM.docx]

**Supplementary Table 2.** Number of the participants involved in the study from each country and geographical region

|  | | Region, No. | | | | | | | | No. |
| --- | --- | --- | --- | --- | --- | --- | --- | --- | --- | --- |
|  |  | Eastern Asia | Western Asia | North and South America | Northern Europe | Western Europe | Southern Europe | Eastern Europe | Others |  |
| Country of residence | Cyprus | 0 | 210 | 0 | 0 | 0 | 0 | 0 | 0 | 210 |
|  | Greece | 0 | 0 | 0 | 0 | 0 | 61 | 0 | 0 | 61 |
|  | Switzerland | 0 | 0 | 0 | 0 | 102 | 0 | 0 | 0 | 102 |
|  | Germany | 0 | 0 | 0 | 0 | 59 | 0 | 0 | 0 | 59 |
|  | Austria | 0 | 0 | 0 | 0 | 60 | 0 | 0 | 0 | 60 |
|  | UK | 0 | 0 | 0 | 25 | 0 | 0 | 0 | 0 | 25 |
|  | Belgium | 0 | 0 | 0 | 0 | 7 | 0 | 0 | 0 | 7 |
|  | Finland | 0 | 0 | 0 | 52 | 0 | 0 | 0 | 0 | 52 |
|  | Spain | 0 | 0 | 0 | 0 | 0 | 46 | 0 | 0 | 46 |
|  | Ireland | 0 | 0 | 0 | 133 | 0 | 0 | 0 | 0 | 133 |
|  | Italy | 0 | 0 | 0 | 0 | 0 | 227 | 0 | 0 | 227 |
|  | Latvia | 0 | 0 | 0 | 395 | 0 | 0 | 0 | 0 | 395 |
|  | France | 0 | 0 | 0 | 0 | 94 | 0 | 0 | 0 | 94 |
|  | Canada | 0 | 0 | 0 | 0 | 0 | 0 | 0 | 14 | 14 |
|  | Colombia | 0 | 0 | 43 | 0 | 0 | 0 | 0 | 0 | 43 |
|  | The Netherlands | 0 | 0 | 0 | 0 | 0 | 0 | 0 | 8 | 8 |
|  | Poland | 0 | 0 | 0 | 0 | 0 | 0 | 30 | 0 | 30 |
|  | Romania | 0 | 0 | 0 | 0 | 0 | 0 | 47 | 0 | 47 |
|  | Hungary | 0 | 0 | 0 | 0 | 0 | 0 | 71 | 0 | 71 |
|  | Demark | 0 | 0 | 0 | 0 | 0 | 0 | 0 | 3 | 3 |
|  | Luxembourg | 0 | 0 | 0 | 0 | 0 | 0 | 0 | 6 | 6 |
|  | Slovenia | 0 | 0 | 0 | 0 | 0 | 0 | 0 | 16 | 16 |
|  | Czech Republic | 0 | 0 | 0 | 0 | 0 | 0 | 0 | 3 | 3 |
|  | Mexica | 0 | 0 | 0 | 0 | 0 | 0 | 0 | 2 | 2 |
|  | Portugal | 0 | 0 | 0 | 0 | 0 | 82 | 0 | 0 | 82 |
|  | Turkey | 0 | 36 | 0 | 0 | 0 | 0 | 0 | 0 | 36 |
|  | USA | 0 | 0 | 89 | 0 | 0 | 0 | 0 | 0 | 89 |
|  | Hong Kong | 207 | 0 | 0 | 0 | 0 | 0 | 0 | 0 | 207 |
|  | Vietnam | 6 | 0 | 0 | 0 | 0 | 0 | 0 | 2 | 8 |
|  | Australia | 0 | 0 | 0 | 0 | 0 | 0 | 0 | 3 | 3 |
|  | Sweden | 0 | 0 | 0 | 0 | 0 | 0 | 0 | 3 | 3 |
|  | Russia | 0 | 0 | 0 | 0 | 0 | 0 | 0 | 1 | 1 |
|  | Montenegro | 0 | 0 | 0 | 0 | 0 | 19 | 0 | 0 | 19 |
|  | Slovakia | 0 | 0 | 0 | 0 | 0 | 0 | 0 | 1 | 1 |
|  | Brazil | 0 | 0 | 0 | 0 | 0 | 0 | 0 | 5 | 5 |
|  | Israel | 0 | 0 | 0 | 0 | 0 | 0 | 0 | 1 | 1 |
|  | Argentina | 0 | 0 | 0 | 0 | 0 | 0 | 0 | 9 | 9 |
|  | South Africa | 0 | 0 | 0 | 0 | 0 | 0 | 0 | 2 | 2 |
|  | Norway | 0 | 0 | 0 | 0 | 0 | 0 | 0 | 1 | 1 |
|  | Philippines | 0 | 0 | 0 | 0 | 0 | 0 | 0 | 1 | 1 |
|  | Pakistan | 0 | 0 | 0 | 0 | 0 | 0 | 0 | 1 | 1 |
|  | New Zealand | 0 | 0 | 0 | 0 | 0 | 0 | 0 | 1 | 1 |
|  | North Macedonia | 0 | 0 | 0 | 0 | 0 | 0 | 0 | 1 | 1 |
|  | Iran | 0 | 0 | 0 | 0 | 0 | 0 | 0 | 1 | 1 |
|  | India | 0 | 0 | 0 | 0 | 0 | 0 | 0 | 3 | 3 |
|  | Iceland | 0 | 0 | 0 | 0 | 0 | 0 | 0 | 1 | 1 |
|  | China | 0 | 0 | 0 | 0 | 0 | 0 | 0 | 1 | 1 |
|  | Chile | 0 | 0 | 0 | 0 | 0 | 0 | 0 | 1 | 1 |
|  | UAE | 0 | 0 | 0 | 0 | 0 | 0 | 0 | 1 | 1 |
|  | Belarus | 0 | 0 | 0 | 0 | 0 | 0 | 0 | 1 | 1 |
|  | Andorra | 0 | 0 | 0 | 0 | 0 | 0 | 0 | 1 | 1 |
|  | Uruguay | 0 | 0 | 0 | 0 | 0 | 0 | 0 | 1 | 1 |
|  | Taiwan | 0 | 0 | 0 | 0 | 0 | 0 | 0 | 1 | 1 |
| Total No. | | 213 | 246 | 132 | 605 | 322 | 435 | 148 | 96 | 2197 |
